# Supplementary material for: The cost of noise: Stochastic punishment falls short of sustaining cooperation in social dilemma experiments
Source: PLoS One. 2022 Mar 2;17(3):e0263028. doi: 10.1371/journal.pone.0263028 (PMC8890652; doi:10.1371/journal.pone.0263028)
Supplement: S1 Text — The details of experimental procedure and supplementary analysis of the experimental data is presented. (PDF) [file pone.0263028.s001.pdf]

Supplementary information for:
The cost of noise: stochastic punishment falls short of sustaining cooperation in social dilemma experiments

Mohammad Salahshour, Vincent Oberhauser, and Matteo Smerlak

Max Planck Institute for Mathematics in the Sciences, Leipzig, Germany

Appendix A: Supplementary information concerning the experiments

1. Experimental design

The experiments were conducted from March to July 2021 using oTree. We recruited participants using the online platform `prolific.co`. The sole reason for exclusion was prior participation in a study. Participants were paid a fixed compensation of £3.75 with the opportunity to earn a bonus of up to £6 based on their income in the game. In total, 384 participants in 96 groups finished the experiment. After removing groups with repeated non-responses in order to eliminate bias, 80 groups remained for the analysis, involving 320 participants from 41 countries (sex ratio $\simeq 2:1$). These are distributed on the experimental conditions in the following way: 21 groups in the control condition, and 20 (resp. 20, 19) groups in the low (resp. medium, high) noise treatment condition.

After accessing the study via study link, the participants were presented with an information sheet to which they had to agree and were asked to identify themselves using their Prolific identification. After that, participants were shown six pages of instructions explaining the game, followed by a set of control questions to ensure their understanding. The content of the information sheet, the instruction pages, and the control questions are listed below.

After correctly completing the control questions, participants were assorted into groups of four. The identity of the other players was concealed, their displayed order randomized in between rounds. They played 22 rounds of a Public Goods Game, the first two rounds being labeled as test rounds, not counting towards the final income. The actual number of rounds was not communicated to the participants to avoid defective behavior in the final rounds.

At the start of the game, participants were endowed with 200 Coins, with each Coin worth £0.01. The game itself was divided into two stages. In the first stage, participants were given 10 Coins, of which they had the choice to contribute any amount to a non-specified group project and

keep the rest for themselves. Each Coin invested in the group project was multiplied by factor 2 and distributed evenly between all group members.

In the second stage, participants were shown the other group members' contributions to the group project and allowed to spend up to 10 Coins per group member to reduce that player's income. The experiment contained four experimental conditions. The treatment conditions differed by the factor used to enhance the amount paid to reduce other players' income. In the control condition, each Coin spent to reduce income was multiplied by three, and the result was subtracted from the punished player's account. The three treatment conditions introduced a noise parameter of varying degrees, with the multiplication factor for each payment being drawn from a continuous uniform distribution for each treatment condition. The distributions all exhibit a mean of 3 but vary widely in range. Distributions with the bounds 2 to 4, 1 to 5, and 0 to 6 were chosen. After seeing the effects of reduction payments and the amount of punishment received by others, the participants proceeded to the next round. At the end of the game, the final income from the game was shown, and participants were asked to answer a number of survey questions that are listed below. Participants that concluded the survey were given a completion link to receive their compensation and bonus via Prolific.

2. Study information sheet

Participants had to agree to the following information to take part in the study.

Thank you for your interest in our experiment. Please read through this information carefully before deciding whether or not you wish to participate.

This experiment is performed as part of a **research project** carried out at the Max Planck Institute for Mathematics in the Sciences (Germany). The purpose of this research is to study how people make economic decisions, both individually and as members of a group. In addition, we are interested in the cultural, social, and individual factors which underlie these decisions.

The experiment is a multiplayer game, which means that you will **play with other participants**. The game will last a fixed number of rounds, and should not last longer than an hour. Unless you withdraw for the experiment before its end, you will receive a **fixed compensation plus a variable bonus** based on your performance in the game.

After the game, you will be asked to share your age, gender, and nationality, as well as general information about your investment and gambling habits. We will not ask for your name or any other information which could be

used to identify you. The data collected will be used for **research purposes**, and may appear in future scientific publications.

You have the right to obtain additional information about the study by contacting the head of the study.
While we hope that you complete our experiment, you will be free to withdraw at any time without prejudice.

By checking the following box you confirm that you understand and accept the terms below:

Study participation:

- I have been informed about the study and its nature, significance, scope and associated risks, and have read and understood this information.
- I have the right to request additional information about the study at any time.
- I hereby declare that I am willing to participate voluntarily in the study.
- I have been notified that I may withdraw from the study at any time without prejudice.
- I have received a copy of the information for test subjects and declaration of consent (online).

Data protection:

- Data collected about me during this study may be used by the Max Planck Institute for Mathematics in the Sciences (MPI-MiS) for the purpose of scientific research.
- The collected data will be recorded in paper or digital formats, stored and scientifically evaluated in a pseudonymous way in compliance with data protection regulations. The security of this database meets the latest standards and is subject to strict system access control.
- The head of the study shall have overall responsibility for the processing of data collected in the course of this study.

3. Experimental instructions

Before the game, participants were given the following instructions:

Instruction Page 1/6

In this experiment, you will play a game and have an opportunity to earn money from it. How much you earn will depend on your performance in the game, and will be paid out to you as a bonus (in addition to the fixed participation fee). Please read these instructions carefully, as they can help you win more in the game.

You have an account in this experiment. At the onset, your account is endowed with **200.0 Coins.** Each Coin is worth **0.01 Pounds** (one penny). Any Coins that you win or lose during the game will be added to your account. Your account balance will be converted to real currency and paid out to you at the end of the experiment.

The game will be played for multiple rounds, with **4 participants**, including you. In each round, you will make a decision and gain or lose Coins based on that decision as well as other players' decisions. Because this is

a multiplayer game, there is a timer on each page, that will show the time left to stay on this page. After reading the instructions you will have the opportunity to play two training rounds with longer timers to familiarize yourself with the game. Each round of the game has **2 stages.** These stages are explained in the next pages. Please press the "Next" button to proceed.

Instruction Page 2/6

First stage: Contribution

In the first stage of each round, each participant will be endowed with 10 Coins. Each participant may invest any amount they choose, from zero up to their whole endowment of 10 Coins, in an unspecified group project. The project is profitable: each 1 Coin invested by all players returns 2 Coins, to be shared equally among all players. Any Coins that you choose not to invest in the group project are added to your account.

Below you can see an example of the page that you will see in the first stage. After deciding about your contribution by filling the decision field on this page, press the "Next" button to proceed to the next page.

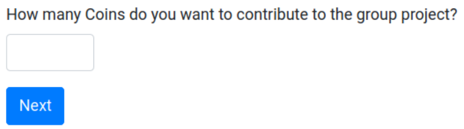

Instruction Page 3/6

Result of the contribution On this page, you will see the result of the first stage. You can see a summary of your contribution to the group project, the total contribution by all group members, the Coins you have kept in your account, your income from the group project, your total income in this round, and your total account balance in the experiment.

Below you can see an example of this page. After seeing this information, please press the "Next" button to proceed to the next page.

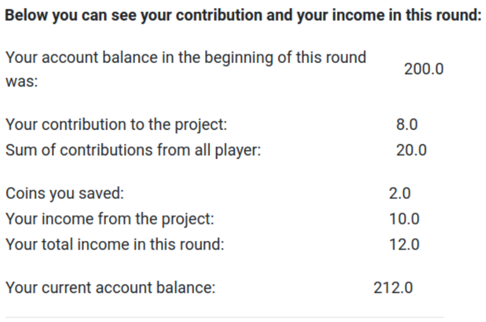

Below you can see your contribution and your income in this round:

Your account balance in the beginning of this round was:	200.0
Your contribution to the project:	8.0
Sum of contributions from all player:	20.0
Coins you saved:	2.0
Your income from the project:	10.0
Your total income in this round:	12.0
Your current account balance:	212.0

Instruction Page 4/6

Second stage: You may reduce other player's income

In the second stage of each round, you will have an opportunity to reduce each other players' income, by paying a cost. You may assign any amount from zero up to 10 Coins, to each other player to reduce their income.

This paragraph was only shown to players in the control condition:
For each Coin assigned to reduce a player's income, **1 Coin will be subtracted from your account, and 3 Coins will be subtracted from the other player's account.**

For the three treatment conditions, the following paragraph with the respective applicable numbers was shown:
For **each Coin** assigned to reduce a player's income, 1 Coin will be subtracted from your account, and an **amount chosen randomly between 0 and 6** (with equal probability) will be subtracted from the other player's account. For example, if you assign 2 Coins to reduce a player's income, the effect may range from 0 Coin to 12 Coins subtracted from the player's account.

After finalizing your decision on how much you want to allocate to reduce each other player's income, please press the "Next" button to confirm your decision and proceed to the next page, where you will see the results of income reductions.

Below you see an example of this page. In each row, you can see how much a player has contributed to the group project and their income in this round. Assign as many Coins as you wish to reduce each player's income by typing the amount of your choice in the box in the player's row. Note that the order with which other players appear on this page is determined randomly in each round.

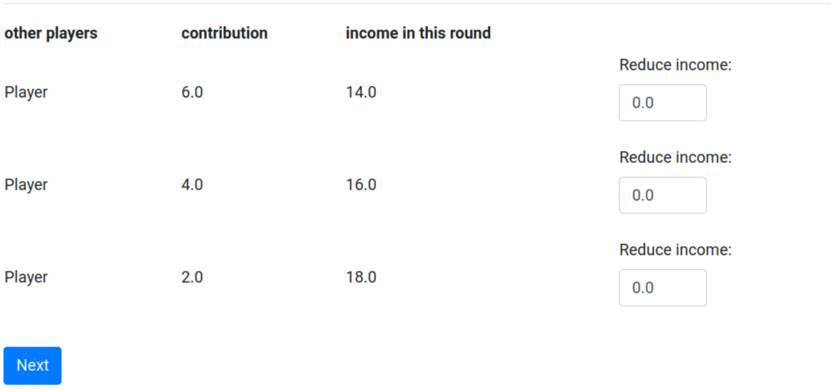

Instruction Page 5/6
On the last page of each round you can see how many Coins have been subtracted from your account by other players in total. You are also informed about your total income in this round.

Your income in a round is determined in the following way:

- **the Coins you did not invest in the project and that were saved in your account,**
- **plus the payoff from the project,**
- **minus the Coins you have assigned to reduce other players' incomes,**
- **minus the Coins others have subtracted from your account.**

Also your current account balance is shown to you. Below this information you see the actual amount of Coins that was subtracted from other players' accounts due to your assignment of Coins. After seeing this information, you will play the next round in the same way as before.

Below you see an example of what this page looks like.

Your income from the first stage:	12.0
The cost you paid to reduce other players' incomes:	3.0
The amount of Coins others payed in this round to reduce your income:	0.0
The amount you were fined in this round:	0.0
Your income this round(including Coins subtracted by other players and cost of reducing other players' incomes):	9.0
Your current bankroll:	209.0

Below, you can see the contribution of each other player, the total amount you payed to reduce this player's income, and their fine recieved:

players	contribution	Coins paid to reduce player's income	Coins substracted from player's account
Player	4.0	**1.0**	**3.0**
Player	2.0	**2.0**	**6.0**
Player	6.0	**0.0**	**0.0**

Next

Instruction Page 6/6

Illustration of a round: This figure shows an example of a round. Try to understand what is happening. It will help you answer the test questions on the following page.

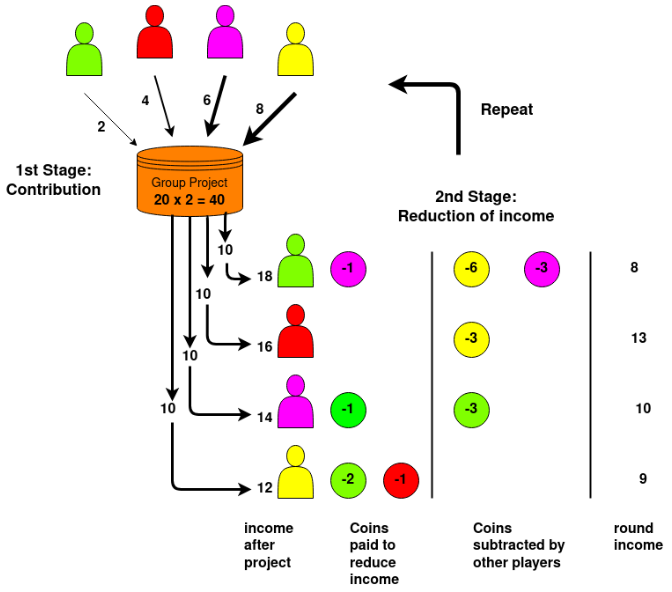

4. Control questions

To proceed to the game, participants had to answer the following control questions correctly:

In order to be able to proceed to the game, you need to answer the following questions. If necessary, you may go back to review the instructions.

Each group member is given an endowment of 10 Coins. Suppose you contribute 10 Coins to the project. All other group members each contribute 10 Coins to the project.
What is your income? (consisting of the project's payoff and the Coins saved in your account):

What is the income of each other group members? (consisting of the project's payoff and the Coins saved in their accounts):

Each group member is given an endowment of 10 Coins. Suppose the other three group members contribute a total of 20 Coins to the project.
What is your income if you contribute 0 Coins to the project? (consisting of the project's payoff and the Coins saved in your account):

What is your income if you contribute 4 Coins to the project? (consisting of the project's payoff and the Coins saved in your account):

Assume you assign 2, 3, and 0 Coins to reduce the three other players' incomes. How much in total will this cost you?

For each Coin you assign to reduce their income, the amount of Coins subtracted from their account is:

Assume the following situation: You invest 4 Coins in the group project. You save 6 Coins in your account. You receive 8 Coins from the group project. 8 Coins from your account are subtracted by others. And you assign 3 Coins to reduce other players' income.
How much will your total income in this round be? (consisting of the project's payoff and the Coins saved in their accounts, minus the Coins assigned to reduce other players' incomes and the Coins subtracted from your account):

5. Survey

After playing 22 rounds of the game players were presented with the following survey:

Please answer the following questions.

What is your nationality?

What is your gender?

What is your age?

On average, do you think you have contributed more, less, or equal than the average contribution in your group? :

On average, do you think you have reduced other players' incomes more, less, or equal than the average in your group? :

On average, do you think other players subtracted from your account more, less, or equal than the average in your group? :

On average, how many Coins do you think you spent on reducing other players' incomes per round? :

On average, how many Coins do you think you contributed to the group project per round? :

If for each Coin assigned to reduce a player's income 3 Coins would have been subtracted from their account, how many Coins do you think you would have spent on reducing other players' incomes on average in each round? :

If for each Coin assigned to reduce a player's income a randomly chosen amount, with an average of 3 Coins, would have been subtracted from their account, how many Coins would you have contributed on average in each round? :

What were your criteria to reduce other players' incomes? Please type your answer in the box below.

Please use this box to tell us about any other comments that you may have.

Appendix B: Supplementary analysis

1. Supplementary analysis of punishment patterns

To study punishment patterns, we begin by plotting the probability that an individual assigns different amounts of punishment, from 0 (not punishing) up to the maximum possible value of 10 Money Units, in the control condition and three different treatments. The probability that an individual does not punish decreases monotonically with the strength of noise. On the other hand, the probability of assigning from 1 up to about 4 Money Units also increases with noise. Assigning higher amounts for punishment occurs with a much lower probability in all conditions.

A breakdown of prosocial and antisocial punishment depending on the level of the sociality of the punishment is provided in Fig. 2. The top panels show the average punishment imposed by a focal individual on a group member with a given contribution difference with respect to the focal individual. The bottom panels show the probability that a focal individual punishes a given contribution difference with respect to her own contribution. In Fig. 2, we plot the increase in the average Money Units that an individual pays to punish a given deviation from her contribution in different stochastic treatments with respect to the control condition.

Inspection of the figures shows that stochasticity increases weak prosocial and antisocial punishment. On the other hand, stochasticity decreases strong prosocial punishment, defined as the punishment of those who contribute 8 or more Money Units less than the punisher. In contrast to the increase in weak prosocial and antisocial punishment, which increases with increasing the level of stochasticity (that is, the difference in weak prosocial and antisocial punishment with respect to the control condition is higher in more stochastic treatments), even a small amount of stochasticity can significantly decrease strong prosocial punishment.

A peculiarity is observed in the case the maximum antisocial punishment. That is when the contribution difference of punisher and punishee equals 10. In this case, punishment probability does not show a significant difference between different stochastic treatments and the control condition. Punishment magnitude, however, is lower in the stochastic treatments compared to the control condition. We note that this case corresponds to full free-riders, those who do not

contribute to the public pool punishing full cooperators, that is, those who contribute the highest amount to the public pool.

2. Regression analyses

a. Regression analysis of group-averaged contributions and payoffs

An ordinary least-square regression analysis of group-average contribution, presented in Table IA, confirms our conclusions: contribution decreases with noise amplitude ($\sigma = 0, 1, 2, 3$ in respectively, the control, low, medium, and high noise groups) across all the treatments (model 1). However, as argued before, the dependence of contributions on noise is mediated by a change in punishment patterns. When we control for the group-average prosocial and antisocial punishment (model 2), the association between contribution and noise ceases to be significant. Instead, contributions show a negative association with antisocial punishment, which—as we will show shortyly—in turn increases with noise. These two facts—noise increases antisocial punishment, antisocial punishment decreases contributions—explains the degraded outcome observed in the noisy treatments.

These effects are confirmed when we model directly the dependence of payoff on contribution and punishment patters within the group. As shown in table IB, payoffs drop with the noise amplitude (model 1), but this is explained by punishment patterns and initial contribution (model 2): a group that starts off more generous and punishes less continues to contribute more—and collect higher payoffs.

b. Censored regression of individual punishment decisions

The results of the Tobit regression analysis of antisocial and prosocial punishment are presented in Tables II and III. Here, the assigned punishment points are used as the dependent variable, separated for the prosocial (Table II) and antisocial (Table III) cases. As the independent variables, we use the punished subject's contribution, the punisher's contribution, punishment received by the punisher at the previous period, period, a dummy variable to take last round effects into account, the average contribution of others in the group (other than the punisher and the punished subjects). A constant is also included in the regression models.

In the case of prosocial punishment, in all the treatments, the punished subject's contribution is negatively related to the punishment points assigned to the subject. However, this relation

is stronger in the control condition, suggesting noise decreases the association between a subject contribution and punishment imposed on the subject. Punisher's contribution shows a positive and strongly significant relation to punishment points assigned only in the control condition. In low stochasticity treatment, this relation becomes weakly significant and non-significant in medium and high stochasticity conditions.

Punishment received by the subject in the previous round has a positive relation to punishment points assigned in high (strongly significant) and medium (significant) stochasticity conditions. A significant relation does not exist in the control condition, and a weakly significant negative correlation is observed in the low stochasticity conditions. These results suggest stochasticity increases revengeful motives behind punishment in the case of prosocial punishment.

Period shows a negative and weakly significant relation to punishment points assigned only in the medium and high stochasticity treatments. This pattern suggests prosocial punishment slightly decreases in later rounds. Besides, the last round dummy variable does not show any significant effect, suggesting that the last round effects are not at work.

Finally, punishment points assigned positively relate to the average contribution of others in the groups in all the treatments. That is, groups where others contribute more exhibit a higher amount of prosocial punishment.

The analysis of antisocial punishment in Table III shows punishment points assigned negatively relate to the punished subject's contribution in the stochastic conditions but not significantly so in the control condition. This suggests under noisy conditions, the more a subject contributes, the less severe the antisocial punishment imposed on the subject.

The punisher's contribution shows a negative and significant relation to punishment points assigned in the control and low and medium stochasticity conditions, but not in the high stochasticity condition. That is, in these conditions, the more subjects contribute, the less antisocially they punish. Punishment received by a subject in the previous round shows a positive and strongly significant relation to punishment points assigned by the subject only in the control and high stochasticity condition.

The effect of the period is overall negative, and its significance varies across treatments, and last round effects are not observed in any of the treatments. Finally, antisocial punishment correlates with the average contribution of others in the group only in the stochastic treatments. That is, in these treatments, the more others in the group contribute, the more prevalent antisocial punishment is.

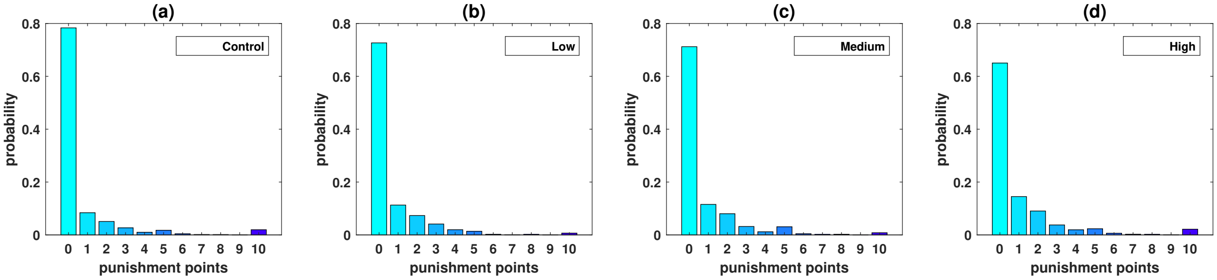

FIG. 1. The probability of assigning 0 up to 10 punishment points. The probability of assigning zeros punishment points (not punishing) monotonically increases with increasing stochasticity, while the probability of punishing from 1 up to approximately 5 punishment points increases with increasing stochasticity.

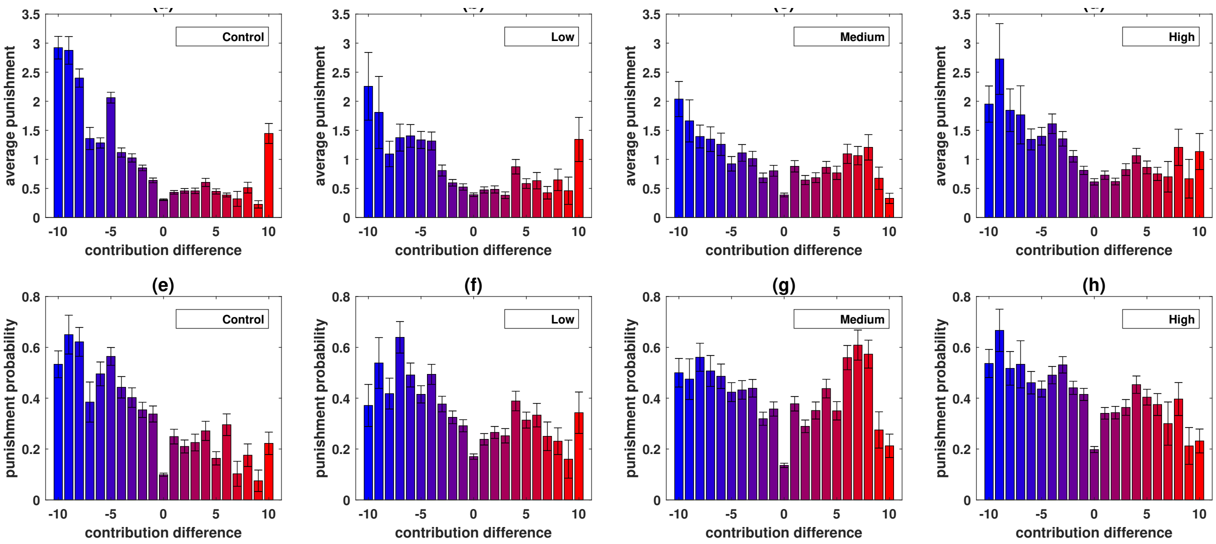

FIG. 2. Average punishment (top) and punishment probability (bottom) for different deviations from ones's own contribution. Both the probability and the magnitude of prosocial punishment decreases in stochastic conditions, While the probability and magnitude of antisocial punishment increases with increasing stochasticity.

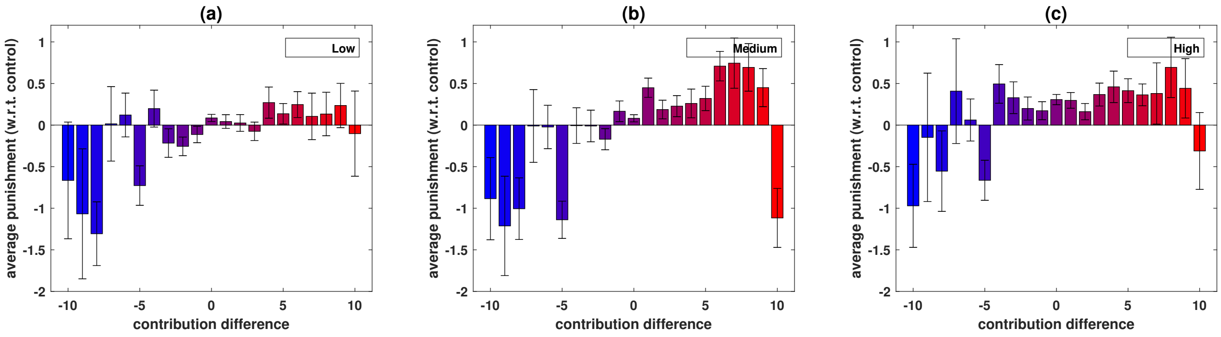

FIG. 3. The increase in the average punishment of a given deviation from the punisher's contribution in stochastic treatments with respect to the control condition.

r

TABLE I. Ordinary least square regression model for contributions (A) and payoffs (B). Group average contributions from period 2 to 20 (A) and group average payoffs from period 1 to 20 (B) are the dependent variable. In Model 1, noise amplitude is used as the dependent variable. In model 2, group average contributions in period 1, group average payoffs, group average antisocial and prosocial punishments are added as independent variables. While contributions and payoffs show a decreasing trend with respect to the noise amplitude (Model 1), controlling for other variables (Model 2) can better explain this pattern.

	A) Dependent variable: Group average contribution	
	Model 1	Model 2
Noise amplitude	-0.38567**(0.1762)	-0.2446 (0.1505)
Group average contribution in period 1	-	0.4194*** (0.0915)
Group average prosocial punishment	-	0.4242 (0.2696)
Group average antisocial punishment	-	-0.9011** (0.3797)
Constant	7.7087***(0.3288)	4.5939*** (0.7266)
Observations	79	79
Adjusted r^2	0.046	0.330
F statistics	4.79	10.6
P-value	0.0317	0.0000

	B) Dependent variable: Group average payoff	
	Model 1	Model 2
Noise amplitude	–1.511*(0.8049)	-0.5149 (0.3903)
Group average contribution in period 1	-	0.7655*** 0.2506
Group average prosocial punishment	-	-2.2926*** (0.7384)
Group average antisocial punishment	-	-8.5938*** (1.0399)
Constant	10.289***(1.5019)	11.814***(1.9898)
Observations	79	79
Adjusted r^2	0.0313	0.755
F statistics	3.52	61.2
P-value	0.0643	0.0000
Note:		*p<0.1; **p<0.05; ***p<0.01

TABLE II. Tobit Model for prosocial punishment separated by treatments. The dependent variable is punishment points assigned. A Tobit model where the dependent variable is bounded between zero and 10 is used.

	Prosocial punishment			
	Dependent variable: Assigned punishment points			
Contributed in t	Control —	Low stochasticity —	Medium stochasticity —	High stochasticity
Punished subject's contribution	-0.7518*** (0.0649)	-0.5528*** (0.0485)	-0.3074*** (0.0442)	-0.3927*** (0.0533)
Punisher's contribution	0.5169*** (0.0829)	0.0809* (0.0562)	-0.0491 (0.0486)	-0.0095 (0.0573)
Punishment points assigned upon at t-1	-0.0008 (0.0414)	-0.0669* (0.0445)	0.0957** (0.0352)	0.2253*** (0.0308)
Period	-0.0140 (0.0287)	-0.0087 (0.0195)	-0.0284* (0.0184)	-0.0343* (0.0219)
last round	0.0762 (0.8319)	-0.0028 (0.5477)	-0.2926 (0.5278)	-0.638 (0.6373)
average contribution of others in group	0.1229** (0.0645)	0.4238*** (0.0483)	0.1541*** (0.0412)	0.3338*** (0.0519)
Constant	-2.3732*** (0.7841)	-1.8437*** (0.4997)	0.0895 (0.4477)	-1.1863* (0.5901)
Observations	1152	1641	1432	1770
Sigma	4.5584	3.491	3.251	4.298
log likelihood	-1398	-1647	-1602	-2156

Note: *p<0.1; **p<0.05; ***p<0.01

TABLE III. Tobit Model for antisocial punishment. The dependent variable is punishment points assigned. A Tobit model where the dependent variable is bounded between zero and 10 is used.

	Antisocial punishment			
	Dependent variable: Assigned punishment point			
Contributed in t	Control —	Low stochasticity —	Medium stochasticity —	High stochasticity
Punished subject's contribution	-0.0241 (0.1280)	-0.1428** (0.0537)	-0.1064** (0.0447)	-0.1383** (0.0487)
Punisher's contribution	-0.2853** (0.1000)	-0.2716*** (0.0475)	-0.1863*** (0.0408)	0.0362 (0.0458)
Punishment points assigned upon at t-1	0.3182*** (0.0579)	-0.0464 (0.0369)	-0.0403 (0.0302)	0.2115*** (0.0245)
Period	-0.0564 (0.0444)	-0.1322*** (0.0187)	-0.0254* (0.0168)	-0.0243 (0.0182)
last round	1.3692 (1.2211)	0.4462 (0.5846)	0.3994 (0.4624)	-0.4347 (0.5454)
average contribution of others in group	0.1258 (0.0996)	0.3268*** (0.0469)	0.2515*** (0.0384)	0.1164** (0.0423)
Constant	-5.1593*** (1.2486)	-0.0082 (0.4573)	-0.0749 (0.4046)	-1.3478** (0.4813)
Observations	1152	1641	1432	1770
Sigma	5.726	3.102	2.919	3.423
log likelihood	-799	-1261	-1479	-1768

Note: *p<0.1; **p<0.05; ***p<0.01
