# Supplementary material for: The cost of noise: Stochastic punishment falls short of sustaining cooperation in social dilemma experiments
Source: PLoS One. 2022 Mar 2;17(3):e0263028. doi: 10.1371/journal.pone.0263028 (PMC8890652; doi:10.1371/journal.pone.0263028)
Supplement: Attachment — Submitted filename: RevieweResponse.pdf [file pone.0263028.s002.pdf]

Responses to review comments

Summary of changes

Two paragraphs are added to the Discussion section based on the referee's suggestions to discuss the effect of the strength of social dilemma, punishment technology, and cross-cultural differences. Eleven references are added to document the additions.

Response to Reviewer N. 1

Reviewer N. 1:
This is a substantially informative and admirably interesting work, which highlights on PGG with punishment scheme from experimental approach. The authors main concern is whether noise of punishment process enhancing or devastating cooperation; and entailing pro- or anti-social punishing intention. Their experimental design was quite intrigued and data analysis in view of statistics was scientifically solid. Visual results are impressive and persuasive, which prove that noisy punishment deteriorates the bolster effect by a punishment scheme. I'm impressed their finding that, despite an individual who punishes defectors from his fair intention in terms of a social norm, a noisy punishment system, more or less, inevitably affects on the result.
Response:
We thank the reviewer for their reading of the manuscript and for their constructive suggestions.
Reviewer N. 1:
Since visuals and text aside the content itself are all well-organized, I would like to suggest this MS as it is to publication. Yet, I would give several comments as below, which are expected to be considered to finalize their MS.

One this is their experimental design. PGG presumes the so-called dilemma weakness parameter; r of 2 while the average amplification factor of punishment (that is denoted by the average ration of fine; Beta, and cost; Alpha) is 3. I wonder why the authors designed (chosen) this particular setting; (r, Ave[Beta/Alpha]) = (2, 3). I definitely believe their result would have a keen sensitivity from the combination of (r, Ave[Beta/Alpha]). Or simply, what happens if bit more severe dilemma situation (less than r = 2) is levied to responders? As well known, PGG can be translated to one of the sub-classes of 2 by 2 Prisoner's Dilemma; Donor & Recipient (D & R) game (reference, e.g.; Difference of reciprocity effect in two coevolutionary models of presumed two-player and multi-player games, Physical Review E 87, 062136, 2013). And if one references to the new concept of Social Efficiency Deficit; SED (reference, e.g.; Social efficiency deficit deciphers social dilemmas, Scientific Reports 10, 16092, 2020), under a bit tough dilemma situation, human's intention or expectation to the scheme of Punishment so as to maintain the social fairness would be altered. Of course, the authors' result from the current experiment could tell noting about this point. Yet, I really love to hear the authors opinion on the point in Discussion part or their provision on future works. When some comment and discussion would be added, the relevant literature as above should be cited.
Response:
A marginal per capita return, r/g, equal to 0.4 and 0.5, and a punishment enhancement factor of 3 are the most common choices used in the literature. So we chose these variables to keep our study comparable to other works. The impact of enhancement factor r on cooperation is studied by some work, and it is suggested that contributions increase with marginal per capita return in public goods experiments. To our knowledge, the impact of r on contributions and punishment in public goods

experiments is less studied. Carpenter (2007) shows that contributions decline with smaller r in this context as well. As the referee notes, these considerations suggest the strength of social dilemma affects humans' behavior in public goods experiments, and it might be reasonable to expect under stronger social dilemmas, noise have a more disruptive effect in establishing social norms. Future experiments can address this question. We have added a paragraph to the Discussion section discussing these considerations.

Regarding the effect of the punishment multiplication factor, experimental evidence has shown that a large enough β is necessary for preventing contributions from falling over time. For instance, Nikiforakis 2008, by examining β, equal to 1, 2, 3, and 4, shows that this only happens for $\beta = 3$ or larger. Other studies seem to draw similar conclusions. Moreover, recently it has been shown theoretically that prosocial punishment can evolve for small punishment multiplication factors (Salahshour 2021). These considerations suggest it is an interesting question to study the effect of β on the effect of noise. It might be reasonable to argue that smaller β noise can be more disruptive for the evolution of social norms and more facilitating for antisocial norms. We have added a paragraph to the discussion to discuss these considerations.

Response to Reviewer N. 2

Reviewer N. 2:
Following the experimental design of Science 319.5868 (2008), and Am. Econ. Rev. 90.4 (2000), the authors studied the influence of noisy punishment to the evolution of cooperation. They found that the contributions decrease and punishment efforts intensify with punishment noise increases. Besides, they also observe that uncertainty causes a rise in antisocial punishment. Totally speaking, they authors do a good job, their results and analysis are beautiful and impressive. I support its acceptance before the authors address the following questions.
Response:
We thank the reviewer for their reading of the manuscript, and for finding the manuscript of interest, and for their constructive suggestions.
Reviewer N. 2:
1. Page 3, Experimental design, the authors say "320 participants from 41 countries played online PGG with punishment in groups of 4 participants". I just curious about whether there are any cross-culture effect regarding to the uncertainty causes a rise in antisocial punishment. As the authors cited in the manuscript, ref. 16 and ref. 18, cultural difference or social norm plays an important role to sustaining cooperation. Since the authors already have data from different countries, I suggest the authors to investigate whether there is a cross-cultural effect regarding the influence of noisy punishment, or the necessary discussions are needed.
Response:
This is an interesting question! For several reasons, our current data do not provide proper evidence for answering this question. Most importantly, our data does not provide nearly enough statistical power. Most of the countries are represented in each treatment by only 1 or 2 participants. The most heavily represented countries are represented by 21, 14, and 11 participants in the $[0, 6]$ treatment, where the stronger statistical power is expected. Furthermore, our data is heavily biased toward participants from European or high-income countries. Past research, for instance, Hermann 2008, does not detect high cultural dependence between high-income countries. But they suggest a high cultural difference in antisocial punishment exists in a broader participant pool which includes low-income countries. Participants from low-income countries are heavily underrepresented in our data. Particularly, we have only one participant from a low-income country in $[0, 6]$ treatment. For these reasons, our data does not generally seem suitable for studying cultural differences.

However, we did looked at this question in our data although it was clear from the outset that only highly speculative evidence can be expected. In the attached figure, contributions, punishment, prosocial and antisocial punishment, and payoffs for three countries that were more heavily represented in the data are plotted. All these are European countries. However, this plot provides highly speculative suggestions that the effect of noise shows cross-cultural differences in the level of punishment, prosocial, and antisocial punishment but not contributions. Obviously, none of these suggestions are even weakly significant. However, this can encourage future studies with a proper design to investigate this question. We have added a discussion to the discussion section mentioning the importance of a cross-cultural study, and discussing some reasons why such a cross-cultural variation might be at work. However, as this analysis presented here is highly speculative, we have not drawn any suggestion from this in the paper.

Reviewer N. 2:

2. Ref 17 and Ref 18 were with same experimental conditions but they were conducted on different countries, the results are totally opposite, what's the behind the reason for this opposite results, is it related with noise? If possible, please write your considerations in Discussion part.

Response:

The authors of Ref. 18 (Wu et. al. 2009) seem to have paid attention to replicating the set-up of Ref. 17 (Dreber et. al. 2008). So, at least based on what comes in the paper, no difference in their setups is apparent. Nor is any source of noise apparent to us in their set-up based on the reading of the paper. Given this, at least based on the reading of the paper it is not obvious that the differences can be attributed to noise, and it seems the argument put forward in ref. [18] to justify the differences in terms of differences in cultural attitude to cooperation and punishment can be a likely explanation.

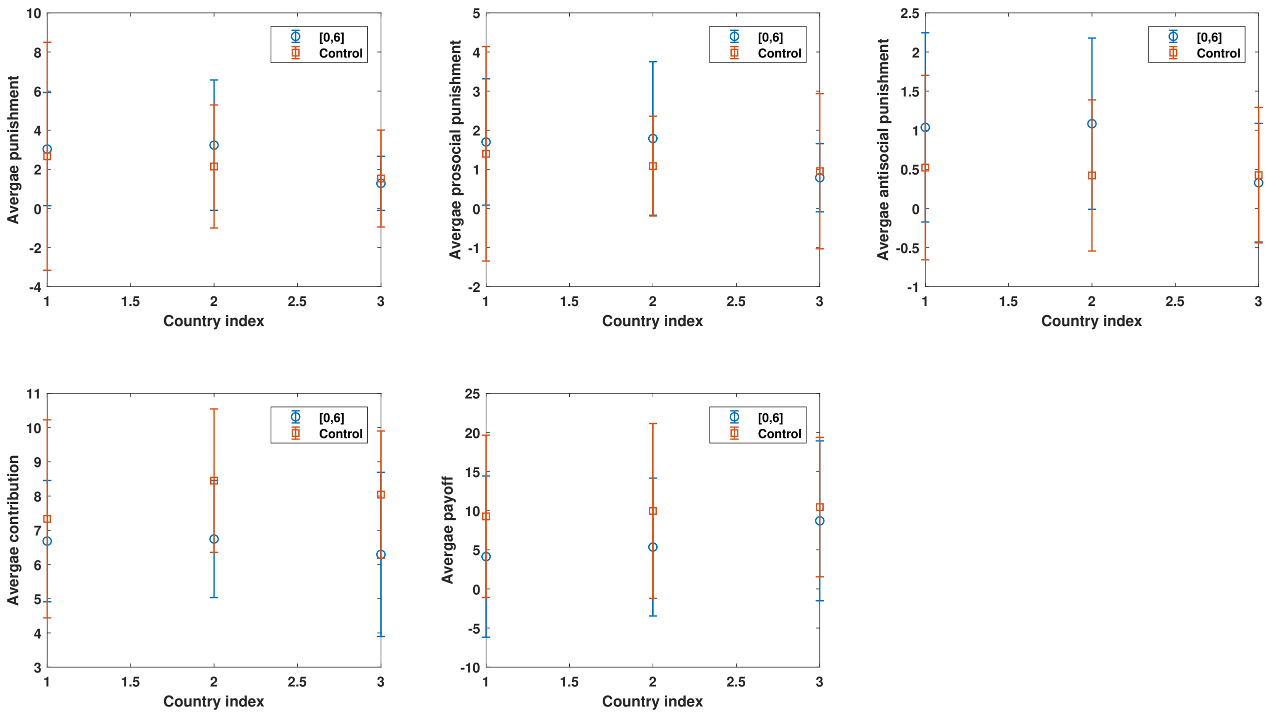

Figure 1: Average punishment, prosocial punishment, antisocial punishment, contributions and payoffs in three for participants from three countries, indexed from 1 to 3 and corresponding to Italy, Poland, and Portugal respectively. Number of participants in Control treatment([0,6] treatment): 9 (11) from country 1, 23 (21) from country 2, and 18 (14) from country 3.
